# Corpos que (re)existem: pensando as experiências de mulheres trans e travestis no cárcere

**DOI:** 10.1590/0102-311XPT262025

**Published:** 2026-06-26

**Authors:** Rayra Pereira Buriti Santos, Gilney Costa Santos, Patricia Constantino

**Affiliations:** 1 Escola Nacional de Saúde Pública Sergio Arouca, Fundação Oswaldo Cruz, Rio de Janeiro, Brasil.

Vanessa Sander, mulher cis, branca, cientista social pela Universidade Federal de Minas Gerais, mestra em Antropologia Social pela Universidade Estadual de Campinas. Autora de *Pavilhão das Sereias: Uma Etnografia dos Circuitos de Criminalização e Encerceramento de Travestis e Transexuais* ^1^, premiado com menção honrosa no Concurso Brasileiro Anpocs de 2025, um relato etnográfico fruto de seu doutorado em Ciências Sociais. Nas 315 páginas organizadas em elementos pré e pós-textuais, e cinco capítulos ilustrados com fotografias, ela materializa o cotidiano social e político da prisão, e daquele que seria seu campo de pesquisa: o Pavilhão das Sereias.

A obra retrata o cotidiano de uma penitenciária masculina de Minas Gerais com ala destinada à população LGBTQIAPN+, que, para ter acesso a uma política considerada mais “segura” e “humanizada”, assina documentos se autodeclarando homossexuais, travestis e/ou trans.

O livro nos convoca a pensar a humanização nas prisões do Brasil. O que seria humanizado, numa unidade prisional em que as práticas de agentes e a estrutura arquitetônica reifica a lógica racial, sexista e de classe? A resposta a essa questão é extremamente complexa, e articula diferentes dimensões da vida social e política do povo brasileiro. Devemos admitir que o sistema prisional é um “Estado de Coisas Inconstitucional” ^2^, por violações massivas aos direitos fundamentais das pessoas privadas de liberdade (PPL).

Minas Gerais está entre os cinco estados de maior extensão territorial do país e, ao alocar todas as pessoas LGBTQIAPN+ em uma única unidade, reforça a prisão como uma instituição total, aprofunda o isolamento e restringe a discussão de gênero no “CIStema” prisional do estado. Tomamos de empréstimo a ideia de “CIStema” de Vergueiro ^3^ para destacar a crítica ao modo como a cisgeneridade organiza o cotidiano da prisão, mantendo privilégios cisgêneros em detrimento das pessoas trans e travestis.

Em pesquisa recente, Santos ^4^ identificou que, no “CIStema prisional” da Bahia, há outra forma de organização de suas unidades, a qual consiste na distribuição dessa população em prisões da capital e do interior do estado, aproximando as PPL de seus territórios e familiares. Apesar disso, persistem diversas fragilidades nesse modelo, que vão desde a falta de ambiência adequada, até mesmo à escassez de políticas de letramento racial, de gênero e sexualidade para agentes prisionais e aprisionados. Em cinco unidades visitadas pela pesquisadora, nenhuma apresentava um “Pavilhão das Sereias” para minimizar os riscos de violências a este público.

Sander argumenta que há uma continuidade entre o “dentro” e “fora” da prisão, entre rua/pista e instituição. Segundo ela, o circuito que envolve esses ambientes e essas pessoas, é um contínuo para a criminalização dos corpos trans e travestis, sendo possível reencontrá-las nas cenas de prostituição e nas prisões. Isso porque as condições de vulnerabilidade e exclusão social lhes impõe tal realidade. Ao analisar o assassinato de uma travesti profissional do sexo em Belo Horizonte, Sander argumenta que o “dentro” e o “fora” da prisão trata esses corpos como abjetos e indesejáveis, neste sentido, apagá-las e/ou afastá-las das relações sociais, seja pelo feminicídio ou pelo aprisionamento, são mecanismos para manutenção da cisheteronormatividade.

O fato é que, neste contexto, o Brasil figura entre os países com a maior população carcerária do mundo, um dos que mais consome pornografia trans e, também, aquele que mais mata essa população ^5^. Sander problematiza a trajetória brasileira de garantias de direito para esse público, chamando atenção para a manutenção de mecanismos punitivistas cisheteronormativos para trans e travestis. A autora observa também que o espaço do “Pavilhão das Sereias”, criado a partir da política pública de segurança em Minas Gerais em 2009, seria um “projeto vitrine” voltado à redução das violências sexuais sofridas por esse público nas prisões.

Nessa perspectiva, a existência de mecanismos de segregação passa a ser compreendido pelos agentes de estado como uma “bomba-relógio”, pois em meio à superlotação e à realidade faccionada do presídio, há pessoas que se declaram homossexuais para “fraudar” o “Pavilhão das Sereias”. Tal espaço representaria os tensionamentos entre as lógicas de proteção e às críticas de inclusão/exclusão dessa população, sendo, portanto, metáfora de um país - Brasil - em que debates sobre gênero e sexualidade são obliterados, e as crises envolvendo a implementação das políticas nas prisões enfrentam resistências institucionais e estruturais.

Sander mergulha nos sentidos, práticas e relações afetivas das travestis do Pavilhão das Sereias e reforça o quanto as redes de afeto são importantes para sobreviver à prisão: “*Aqui travesti é sereia! E olha que é difícil se manter feminina aqui*” (p. 150). Mesmo em meio às precariedades, a prisão masculina se apresenta como espaço em que são desejadas e possibilita desenvolver relações afetivas que facilitem adquirir recursos para sobreviver ao “CIStema”.

Para Sander, o corpo pode ser uma agência, isto é, mecanismo de troca econômica-afetiva, um lugar ambivalente, como o corpo híbrido de uma sereia. Nesse processo, as revistas íntimas/vexatórias marcam esses corpos a partir da produção de constrangimentos e contestação do gênero das internas, seja por terem que se despir e agachar na presença das PPL ou pela resistência das agentes prisionais (cis) em operar a política de revista sob a justificativa de terem seus direitos humanos violados. Diante das vulnerabilidades sociais enfrentadas pela população LGBTQIAPN+ e os riscos à vida enquanto PPL, o Supremo Tribunal Federal (STF) foi provocado a garantir às mulheres trans e travestis o “*direito de opção pelo cumprimento de pena em unidades prisionais femininas ou masculinas, no último caso, em alas específicas, que lhes garanta a segurança*” ^6^ (p. 1), contudo, as violações ainda são reais.

Como uma obra que dialoga com seu tempo, Sander recupera as memórias da experiência das PPL no Pavilhão das Sereias durante a pandemia de COVID-19, em que visitas presenciais foram suspensas e a comunicação com familiares se deu por meio de cartas que denunciavam degradação das condições prisionais, superlotação do Pavilhão e o aumento de suicídios nesse período. Talvez a mensagem central do texto de Sander consista em mostrar que a garantia de direitos às mulheres trans e travestis, apesar dos avanços do debate interseccional, ainda hoje é objeto de disputas dentro e fora das prisões. A bem da verdade, precisamos lembrar que, em contextos sociais e políticos conservadores, os riscos de retrocessos e de apagamento das pautas de direitos humanos tornam-se ainda mais plausíveis. Vimos isso acontecer recentemente no Brasil, o que torna extremamente importante a participação de movimentos sociais pró-direitos humanos e antipunitivistas para manter a pauta no primeiro plano das discussões sociopolíticas.

Registre-se, também, a relevância social e política do texto de Sander para análise e reflexão das condições das mulheres trans e travestis na prisão, que envolve gênero, violência, luta por políticas públicas e as dinâmicas de poder no ambiente prisional que, em geral, invisibiliza e silencia esta população. Mulheres trans e travestis vivenciam realidades diversas nas prisões brasileiras, e o Pavilhão das Sereias, ou melhor, o direito à ala específica, não é uma realidade em todas as unidades prisionais masculinas que resguardam esse público. Indicamos a leitura do livro por reconhecer a necessidade de estudos que coloquem em perspectiva as relações sociais de gênero, raça e classe nas prisões, para o fortalecimento da política e ações de cuidados à saúde das mulheres trans e travestis nas prisões.
